# CXCL16-positive dendritic cells enhance invariant natural killer T cell-dependent IFNγ production and tumor control

**DOI:** 10.1080/2162402X.2016.1160979

**Published:** 2016-04-22

**Authors:** Linnea Veinotte, Simon Gebremeskel, Brent Johnston

**Affiliations:** a Department of Microbiology & Immunology, Dalhousie University, Halifax, Nova Scotia, Canada; b Beatrice Hunter Cancer Research Institute, Halifax, Nova Scotia, Canada; c Department of Pediatrics, Dalhousie University, Halifax, Nova Scotia, Canada; d Department of Pathology, Dalhousie University, Halifax, Nova Scotia, Canada

**Keywords:** Cell activation, chemokines, cytokines, dendritic cells, natural killer T cells

## Abstract

Crosstalk interactions between dendritic cells (DCs) and invariant natural killer T (iNKT) cells are important in regulating antitumor responses elicited by glycolipid antigens. iNKT cells constitutively express the chemokine receptor CXCR6, while cytokine-activated DCs upregulate the transmembrane chemokine ligand, CXCL16. This study examined the co-stimulatory role of CXCR6/CXCL16 interactions in glycolipid-dependent iNKT cell activation and tumor control. Spleen and liver DCs in wild-type mice, but not iNKT cell deficient (Jα18^−/−^) mice, transiently upregulated surface CXCL16 following *in vivo* administration of the glycolipid antigen α-galactosylceramide. Recombinant CXCL16 did not directly induce iNKT cell activation *in vitro* but enhanced interferon (IFN)-γ production when mouse or human iNKT cells were stimulated with plate-bound anti-CD3. Compared with glycolipid-loaded CXCL16^neg^ DCs, CXCL16^hi^ DCs induced higher levels of IFNγ production in iNKT cell cultures and following adoptive transfer *in vivo*. The number of IFNγ^+^ iNKT cells and expansion of T-bet^+^ iNKT cells were reduced *in vivo* when CXCL16^−/−^ DCs were used to activate iNKT cells. Enhanced IFNγ production *in vivo* was not dependent on CXCR6 expression on natural killer (NK) cells. Adoptive transfer of glycolipid-loaded CXCL16^hi^ DCs provided superior protection against tumor metastasis compared to CXCL16^neg^ DC transfers. Similarly, wild-type DCs provided superior protection against metastasis compared with CXCL16^−/−^ DCs. These experiments implicate an important role for CXCR6/CXCL16 interactions in regulating iNKT cell IFNγ production and tumor control. The selective use of CXCL16^hi^ DCs in adoptive transfer immunotherapies may prove useful for enhancing T helper (Th) type 1 responses and clinical outcomes in cancer patients.

## Abbreviations

7-AAD: 7-aminoactinomycin D
α-GalCer: α-galactosylceramide
APC: allophycocyanin
BMDC: bone marrow derived dendritic cell
DC: dendritic cell
ELISA: enzyme-linked immunosorbent assay
FITC: fluorescein isothiocyanate
GM-CSF: granulocyte-macrophage colony-stimulating factor
IFNγ: interferonγ
iNKT cell: invariant natural killer T cell
IL: interleukin
i.v.: intravenous
LPS: lipopolysaccharide
MHC: major histocompatibility complex
NK cell: natural killer cell
PE: phycoerythrin
TCR: T cell receptor
Th: T helper
TNF: tumor necrosis factor

## Introduction

Invariant iNKT cells are a rare population of lipid-reactive T lymphocytes that regulate innate and adaptive immunity. Mice lacking iNKT cells exhibit increased susceptibility to infectious agents and tumor development.^1^ In contrast to conventional T cells, iNKT cells express an invariant Vα14Jα18 T cell receptor (TCR) rearrangement in mice (Vα24Jα18 in humans) that allows recognition of pathogen-associated, tumor-derived, and stress-induced glycolipids presented via the major histocompatibility complex (MHC)-like molecule CD1d.^2,3^ α-galactosylceramide (α-GalCer) and synthetic derivatives are potent activators of iNKT cells,^4-6^ and can prevent tumor development and block experimental metastasis in animal models.^4-10^ The ability of iNKT cells to promote a strong Th 1 response is critical for tumor control as iNKT cell-dependent protection from metastasis is lost in IFNγ^−/−^ mice.^10,11^ Furthermore, elevated IFNγ responses have been associated with prolonged survival in lung cancer patients receiving iNKT cell-targeted immunotherapy via glycolipid-loaded DCs.^12^

Optimal iNKT cell responses to glycolipid antigens require co-ordinated interactions with antigen-presenting cells. DCs present glycolipid antigens to iNKT cells via CD1d, leading to rapid generation of interleukin (IL)-4 and IFNγ,^13,14^ and upregulation of CD40L.^15^ IFNγ increases surface expression of co-stimulatory CD80 and CD86 on DCs,^16^ while CD40L/CD40 interactions induce IL-12 production from DCs.^15,17^ In turn, IL-12 stimulates additional IFNγ production from iNKT cells^17^ and NK cells.^18^ Co-ordinated interactions between iNKT cells and DCs facilitate tumor clearance by mediating the downstream recruitment and activation of effector NK cells,^11,19^ and T cells.^16,20,21^

The chemokine receptor CXCR6 is highly expressed on mouse and human iNKT cells^22-24^ and plays important roles in iNKT cell development, maturation, homeostatic distribution, and glycolipid-induced effector responses.^4,25,26^ Defects in iNKT cell homeostasis and activation have been reported in mice lacking CXCR6 or the cognate ligand CXCL16,^25,26,27^ a chemokine that can be generated in both soluble and transmembrane forms.^28-31^ As transmembrane CXCL16 is upregulated on DCs following activation with lipopolysaccharide (LPS) or cytokines,^23,27,28^ we examined the possibility that CXCL16 may be upregulated during antigenic glycolipid presentation and provide co-stimulatory signals that influence iNKT cell cytokine production and antitumor responses.

Both CXCR6^−/−^ and CXCL16^−/−^ mice exhibit enhanced tumor growth and metastasis in experimental models.^4,27^ However, previous *in vivo* studies could not differentiate whether CXCR6/CXCL16 plays a direct co-stimulatory role in iNKT cell activation as knockout mice have reduced iNKT cell numbers, and impairments in iNKT cell development and maturation.^25-27^ To overcome the influence of iNKT cell defects in CXCR6^−/−^ and CXCL16^−/−^ mice, we used an adoptive DC-based immunotherapy approach to examine the role of CXCR6/CXCL16 interactions in regulating the responses of wild-type iNKT cells. Transfer of glycolipid-loaded CXCL16^hi^ DCs into mice containing wild-type iNKT cells led to enhanced IFNγ responses compared to the delivery of CXCL16^neg^ or CXCL16^−/−^ DCs. Furthermore, glycolipid-loaded CXCL16^hi^ or CXCL16^+/+^ DCs provided enhanced protection from tumor metastasis compared to CXCL16^neg^ or CXCL16^−/−^ DCs. These findings reveal an important role for CXCR6/CXCL16 interactions in regulating iNKT cell function *in vivo* and provide pre-clinical data that support the examination of glycolipid-loaded CXCL16^hi^ DCs in iNKT cell-targeted adoptive transfer therapies for cancer patients.

## Results

### DCs upregulate CXCL16 during crosstalk interactions with iNKT cells

**Figure 1.**
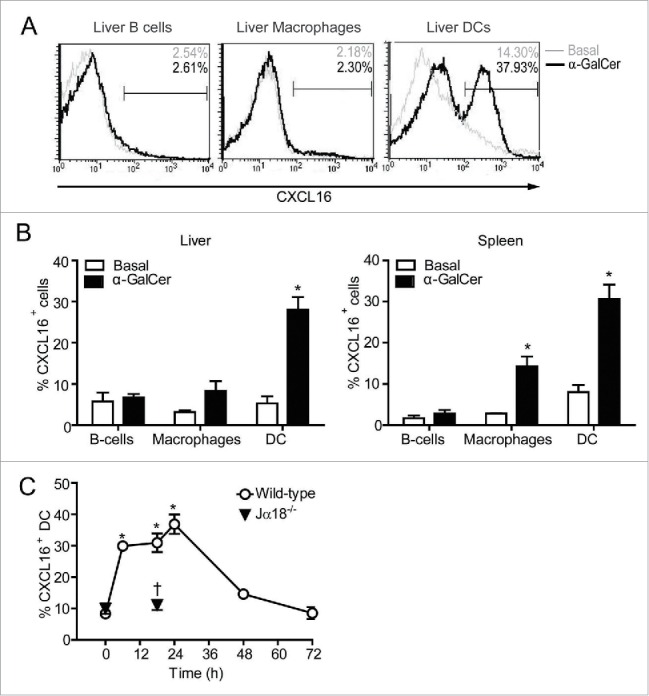
CXCL16 expression on DCs, B cells and macrophages following iNKT cell stimulation with α-GalCer. Spleen and liver cells were harvested 0–72 h after mice were treated intraperitoneally with the glycolipid α-GalCer (4 μg). CXCL16 expression was analyzed on different immune cells by flow cytometry. (A) Representative histograms show CXCL16 expression on liver DCs (CD11c^+^ MHCII^+^), B cells (CD19^+^ B220^+^) and macrophages (CD11b^+^ F4/80^+^) under basal conditions and 18 h after α-GalCer treatment. (B) Aggregate data show CXCL16 expression on liver and spleen cell populations under basal conditions and 18 h after α-GalCer treatment (n = 3–8 per group). (C) Time course of CXCL16 expression on splenic DCs from wild-type and Jα18^−/−^ mice following glycolipid stimulation. (n = 4 per time point). *p < 0.05 compared to baseline. †*p* < 0.05 compared to wild-type.

Human and mouse iNKT cells express high levels of the chemokine receptor CXCR6.^22,24^ CXCL16 is one of only two known chemokines that can be generated as a transmembrane protein,^28,29^ and is upregulated on the surface of activated antigen-presenting cells.^27-29^ This suggests a potential role for CXCR6/CXCL16 in the co-stimulation of iNKT cells. However, little is known about the regulation of CXCL16 during iNKT cell-antigen-presenting cell interactions. As CXCL16 is upregulated spontaneously on human and mouse DCs during *in vitro* culture (ref. 33 and data not shown), we examined regulation of CXCL16 expression on antigen-presenting cells *in vivo*. In control mice, less than 10% of DCs, macrophages and B cells expressed surface CXCL16. After glycolipid administration, the frequency of CXCL16^hi^ DCs increased in the spleen and liver (Figs. 1A–B). Splenic macrophages exhibited a smaller increase in the frequency of cells expressing CXCL16, whereas B cells and liver macrophages did not upregulate CXCL16. We therefore focused our studies on DC-iNKT cell co-stimulatory interactions. CXCL16 expression was upregulated on DCs as early as 6 h following α-GalCer treatment, peaked at 18–24 h, and returned to baseline levels by 48–72 h (Fig. 1C). Upregulation was dependent on the presence of iNKT cells as CXCL16 expression did not increase on DCs in iNKT cell-deficient (Jα18^−/−^) mice treated with α-GalCer (Fig. 1C). This indicates that uptake, processing, and loading of glycolipids onto CD1d is not sufficient to upregulate CXCL16 expression on DCs.

### Recombinant CXCL16 co-stimulates iNKT cells for enhanced IFNγ production

**Figure 2.**
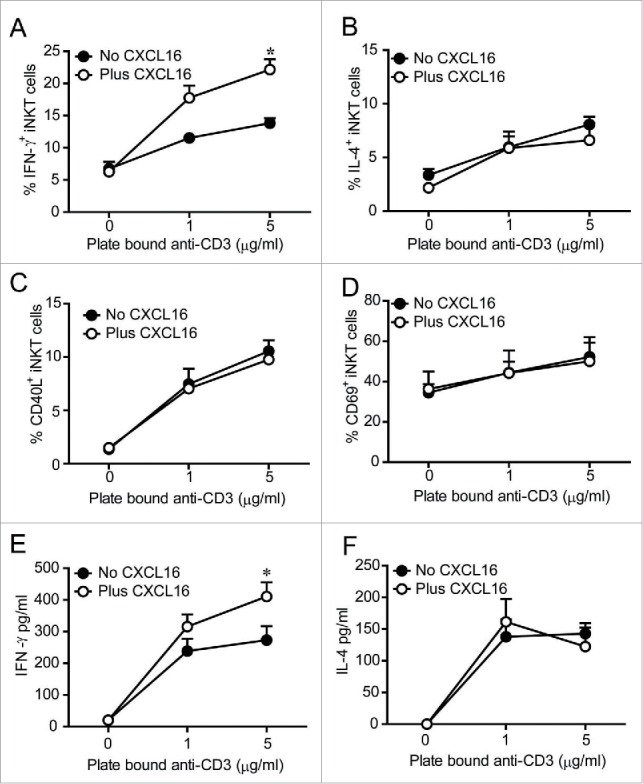
*In vitro* iNKT cell activation in the presence and absence of CXCL16. Liver mononuclear cells were cultured for 2 h in wells coated with 0, 1 or 5 μg/mL anti-CD3, with or without 100 ng/mL of recombinant CXCL16. iNKT cells (CD1d tetramer^+^ TCRβ^+^) were stained to examine intracellular (A) IFNγ and (B) IL-4 production, and cell surface expression of (C) CD40L and (D) CD69 by flow cytometry (n = 3 per group). Sorted human iNKT cells (5 × 10^4^ TCRβ^+^Vα24Jα18^+^) were cultured in wells coated with 0, 1 or 5 μg/mL anti-CD3, with or without 100 ng/mL of recombinant CXCL16. After 24 h supernatants were harvested to measure (E) IFNγ and (F) IL-4 production (n = 5 healthy donors). *p < 0.05 compared with anti-CD3 alone.

After demonstrating that glycolipid-induced CXCL16 upregulation is dependent on iNKT cells, we investigated the co-stimulatory role for CXCL16 in iNKT cell activation. Recombinant CXCL16 on its own did not induce intracellular cytokine production in cultured liver iNKT cells (Figs. 2A–B). However, in the presence of plate-bound anti-CD3, the frequency of iNKT cells staining for intracellular IFNγ production was increased when CXCL16 was present (Fig. 2A). In contrast, anti-CD3-induced intracellular IL-4 production was not altered by CXCL16 (Fig. 2B). The difference in IFNγ staining was not due to a general increase in iNKT cell activation as similar increases in the frequency of CD40L^+^ and CD69^+^ iNKT cells were observed following anti-CD3 activation with and without CXCL16 (Figs. 2C–D). Mean fluorescence intensity of CD40L and CD69 expression increased on CD3-stimulated cells, but was not different in the presence or absence of CXCL16 (data not shown). Consistent with CXCL16-mediated enhancement of IFNγ production in mouse iNKT cells, primary human iNKT cell lines produced higher levels of IFNγ when stimulated by anti-CD3 and CXCL16 versus anti-CD3 alone (Fig. 2E). Production of IL-4 by activated human iNKT cells was not altered by CXCL16 (Fig. 2F). Collectively, these findings suggest that CXCL16 co-stimulation enhances IFNγ production.

### CXCL16 expression on DCs enhances iNKT cell IFNγ production *in vitro*

**Figure 3.**
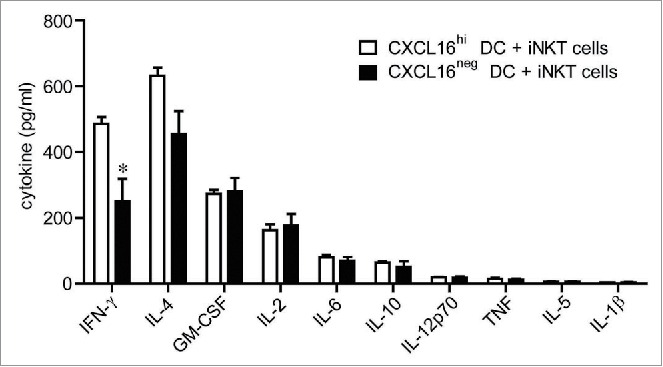
*In vitro* cytokine responses of iNKT cells stimulated with glycolipid-loaded CXCL16^hi^ or CXCL16^neg^ DCs. CD11c^+^ DCs were enriched from the spleen by magnetic sorting and loaded overnight with α-GalCer (200 ng/mL). DCs were sorted into CXCL16^hi^ and CXCL16^neg^ subsets and incubated with sorted iNKT cells (CD1d-tetramer^+^ TCRβ^+^) for 24 h, at a ratio of 1:2, DCs to iNKT cells. Cytokine levels in culture supernatants were examined using a cytokine array (n = 5–6 per group). *p < 0.05 compared with CXCL16^hi^ DCs.

Since recombinant CXCL16 enhanced IFNγ production from anti-CD3-activated iNKT cells, we examined whether CXCL16-expressing DCs could also enhance iNKT cell cytokine production. To dissociate the role of CXCR6/CXCL16 interactions during iNKT cell activation from the developmental and functional defects in CXCR6^−/−^ and CXCL16^−/−^ iNKT cells,^26,27^ we examined the ability of wild-type CXCL16^hi^ and CXCL16^neg^ DCs to activate wild-type iNKT cells. Glycolipid-loaded DCs were mixed with sorted iNKT cells at a ratio of 1:2, and supernatant cytokine levels were analyzed 24 h later by cytokine array. iNKT cells cultured with CXCL16^hi^ DCs produced more IFNγ than those cultured with CXCL16^neg^ DCs (Fig. 3). The levels of granulocyte-macrophage colony-stimulating factor (GM-CSF), IL-2, IL-4, IL-6, IL-10, IL-12p70 and tumor necrosis factor (TNF) were not significantly different between cultures containing CXCL16^hi^ DCs versus CXCL16^neg^ DCs. IL-5 and IL-1β were not generated at levels above the detection threshold of the assay (Fig. 3). Therefore, *in vitro* activation by CXCL16^hi^ DCs appears to selectively induce enhanced IFNγ production by iNKT cells.

### Wild-type CXCL16^hi^ DCs enhance iNKT cell IFNγ production *in vivo*

**Figure 4.**
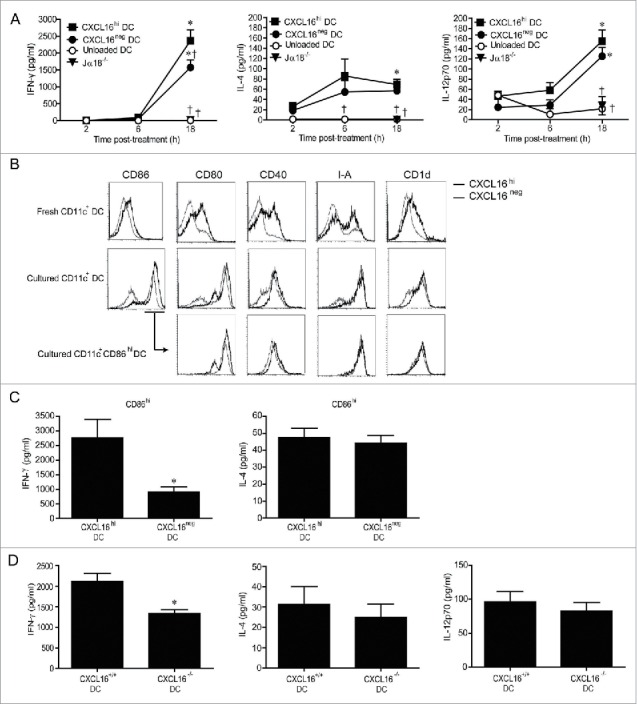
*In vivo* cytokine responses following adoptive transfer of α-GalCer-loaded CXCL16^hi^ or CXCL16^neg^ DCs. CD11c^+^ DCs were enriched from splenocytes by magnetic sorting and loaded overnight with α-GalCer (200 ng/mL). DCs were sorted into CXCL16^hi^ and CXCL16^neg^ subsets and injected i.v. into wild-type or Jα18^−/−^ mice. Unloaded DCs and Jα18^−/−^ mice were used as controls. Serum cytokine levels were analyzed via a multiplex cytokine array. (A) IFNγ, IL-4, and IL-12p-70 levels were measured at 2, 6 and 18 h after DC transfer (n = 5–10 per group). *p < 0.05 compared with 2 h time point, †*p* < 0.05 compared to CXCL16^hi^ DC. (B) Representative plots showing expression of CD86, CD80, CD40, I-A, and CD1d on CXCL16^hi^ DCs and CXCL16^neg^ DCs following isolation and after overnight culture. Receptor expression is also shown for the CD86^hi^ gated population. (C) Serum levels of IFNγ and IL-4 were measured by ELISA 18 h after adoptive transfer of α-GalCer-loaded CD86^hi^ CXCL16^hi^ or CD86^hi^ CXCL16^neg^ DCs. (n = 4 per group). *p < 0.05 compared with CXCL16^hi^ DC. (D) Serum IFNγ, IL-4, and IL-12p70 levels were measured by ELISA 18 h after adoptive transfer of splenic CXCL16^+/+^ or CXCL16^−/−^ DCs (n = 6 per group). *p < 0.05 compared to CXCL16^+/+^ DCs.

We next examined whether CXCL16^hi^ DCs enhanced IFNγ production following glycolipid activation *in vivo*. Glycolipid-loaded splenic CXCL16^hi^ DCs, CXCL16^neg^ DCs, or unloaded DCs were injected intravenously (i.v.) into wild-type mice. iNKT cell-deficient Jα18^−/−^ mice received CXCL16^hi^ DCs as a control to confirm that the cytokines measured in these experiments resulted from iNKT cell activation. At 18 h following DC transfer, higher serum IFNγ levels were observed in mice that received CXCL16^hi^ DCs compared to mice that received CXCL16^neg^ DCs (Fig. 4A). IL-4 levels were not significantly different between mice receiving glycolipid-loaded CXCL16^hi^ or CXCL16^neg^ DCs (Fig. 4A). Levels of IL-12p70 were also equivalent, suggesting that differences in IFNγ levels were not due to altered IL-12 production (Fig. 4A). This is further supported by the observation that the basal level of IL-12p70 production by unloaded DCs was not sufficient to induce IFNγ production *in vivo*. LPS treatment activates DCs and upregulates CXCL16 expression,^27^ but adoptive transfer of LPS-pulsed DCs did not increase IFNγ levels in the serum (data not shown). This suggests that LPS treatment did not upregulate endogenous glycolipid ligands in DCs, and confirms that CXCL16 on its own cannot stimulate iNKT cell responses. Systemic delivery of recombinant mouse CXCL16 also failed to induce iNKT cell activation *in vivo* (data not shown).

Phenotypic analysis of splenic DCs revealed that freshly isolated CXCL16^hi^ and CXCL16^neg^ DCs differed in their expression of CD1d, MHC class II (I-A), and co-stimulatory molecules (Fig. 4B). Both DC subsets upregulated co-stimulatory molecules during overnight culture, but CXCL16^hi^ DCs tended to express higher levels of CD80, CD86, and CD40. These phenotypic differences were normalized when gating on the CD86^hi^ subpopulations of CXCL16^hi^ and CXCL16^neg^ DCs (Fig. 4B). Therefore, adoptive transfers were repeated using CD86^hi^ subsets from both DC populations to ensure that differences in IFNγ production were not due to other phenotypic differences between the DCs. Adoptive transfers of CD86^hi^ CXCL16^hi^ DCs induced more IFNγ than CD86^hi^ CXCL16^neg^ DCs (Fig. 4C). Levels of IL-4 were equivalent, confirming that CXCL16/CXCR6 signaling is important for optimal IFNγ production following iNKT cell stimulation (Fig. 4C). To verify the role of CXCL16 in iNKT cell co-stimulation, transfers were repeated using splenic DCs isolated from wild-type and gene-targeted CXCL16^−/−^ mice (Fig. 4D). Wild-type DCs elicited increased IFNγ responses compared to CXCL16^−/−^ DCs. There were no differences in the levels of IL-4 or IL-12p70 induced by wild-type and CXCL16^−/−^ DCs (Fig. 4D). A similar difference in IFNγ production was observed when bone marrow derived dendritic cells (BMDCs) from wild-type versus CXCL16^−/−^ mice were used instead of splenic DCs (data not shown).

### CXCL16^+/+^ DCs increase the number of IFNγ^+^ and T-bet^+^ iNKT cells

**Figure 5.**
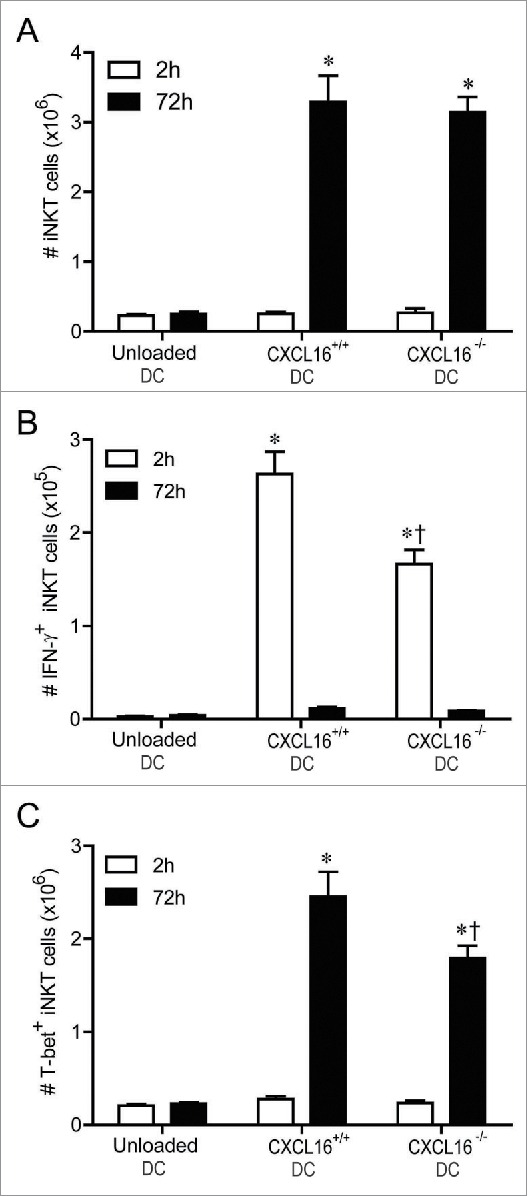
*In vivo* profiling of intracellular IFNγ and T-bet expression in iNKT cells following adoptive transfer of α-GalCer-loaded CXCL16^+/+^ or CXCL16^−/−^ DCs. Mice were stimulated by adoptive transfer of α-GalCer-loaded CXCL16^+/+^ or CXCL16^−/−^ BMDCs. The number of (A) total iNKT cells, (B) IFNγ^+^ iNKT cells and (C) T-bet^+^ iNKT cells was examined by flow cytometry 2 h or 72 h later (n = 3–6 per group). *p < 0.05 compared to unloaded DCs, †*p* < 0.05 compared to CXCL16^+/+^ DCs.

It was not clear whether the CXCL16-mediated enhancement of serum IFNγ levels was due to a selective activation and/or expansion of an iNKT cell subset that produces high levels of IFNγ. iNKT cell expansion was similar in mice receiving α-GalCer loaded CXCL16^+/+^ or CXCL16^−/−^ DCs (Fig. 5A). However, mice receiving CXCL16^+/+^ DCs had an increased number of IFNγ expressing iNKT cells 2 h after stimulation (Fig. 5B). This increase in IFNγ-producing iNKT cells returned to baseline by 72 h following stimulation (Fig. 5B). Moreover, T-bet^+^ iNKT cells expanded to a greater extent by 72 h after transfer of CXCL16^+/+^ versus CXCL16^−/−^ DCs (Fig. 5C). These data suggest that α-GalCer loaded CXCL16^+/+^ DCs enhance polarization and expansion of IFNγ-producing iNKT cells.

### CXCR6^+^ NK cells do not enhance serum IFNγ levels

**Figure 6.**
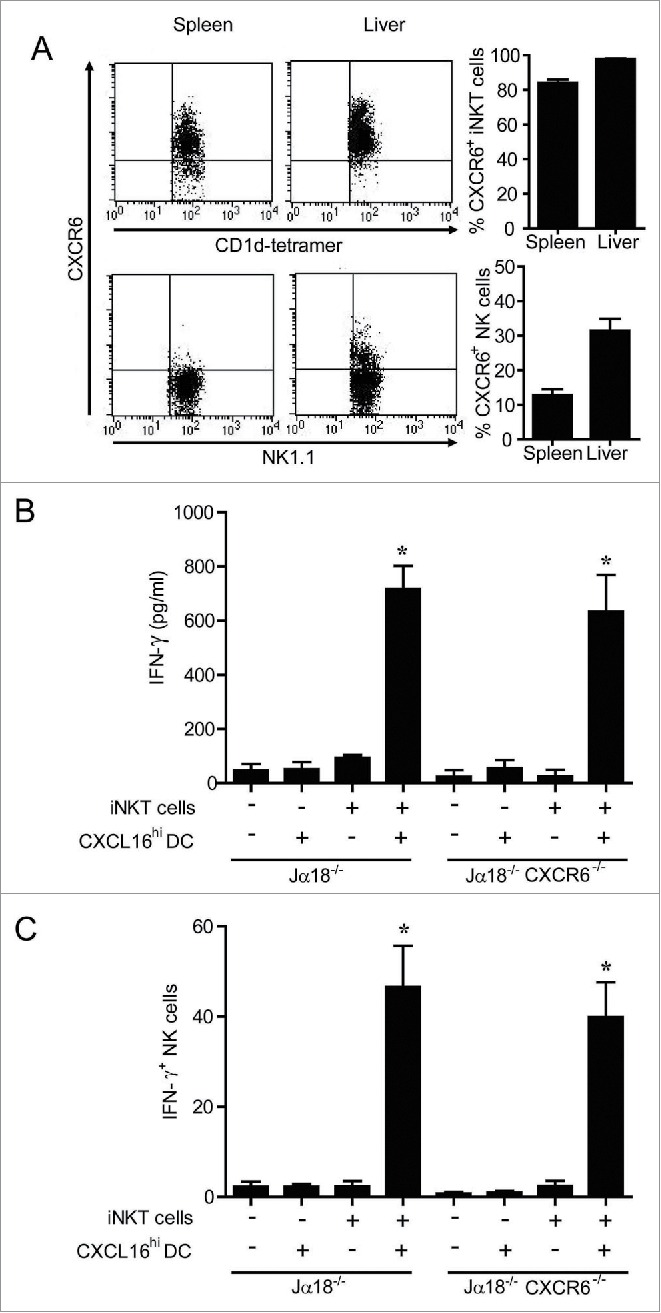
Expression and role of CXCR6 in NK cell transactivation. (A) Surface expression of CXCR6 was examined on NK cells (NK1.1^+^ TCRβ^−^) and iNKT cells (CD1d-tetramer^+^ TCRβ^+^) isolated from spleen and liver using a chimeric CXCL16-Fc construct (n = 4 per group). (B and C) Expanded iNKT cells (CD1d tetramer^+^ TCRβ^+^) from wild-type donor mice were adoptively transferred (i.v. 1 × 10^7^) into recipient Jα18^−/−^ or Jα18^−/−^ CXCR6^−/−^ mice. Twenty-four hours later, control and iNKT cell-reconstituted mice were stimulated with α-GalCer-loaded CXCL16^hi^ BMDCs or unloaded control DCs (i.v. 2 × 10^5^). (B) Serum cytokine levels and (C) NK cell intracellular IFNγ staining were examined 18 h following stimulation (n = 3 per group). *p < 0.05 compared to no DC stimulation.

NK cells are stimulated to produce IFNγ downstream of α-GalCer-induced iNKT cell stimulation.^8,9,19^ Since ∼12% of splenic NK cells and ∼31% of hepatic NK cells express CXCR6 (Fig. 6A), and CXCR6^+^ NK cells have been implicated in antigen-specific anti-viral responses,^34^ it was important to examine the potential contribution of CXCR6/CXCL16 signaling in NK cells to the IFNγ responses induced by transfer of glycolipid-loaded DCs. iNKT cell deficient Jα18^−/−^ and CXCR6^−/−^ Jα18^−/−^ double knockout mice were reconstituted with wild-type iNKT cells 24 h prior to administration of α-GalCer-loaded CXCL16^hi^ DCs. iNKT cell transfer rescued IFNγ production equally in Jα18^−/−^ and CXCR6^−/−^ Jα18^−/−^ mice (Fig. 6B), demonstrating that CXCR6 on NK cells (and other endogenous cells) did not elicit enhanced IFNγ production in response to glycolipid-loaded CXCL16^hi^ DCs. There were no defects in NK cell transactivation as the frequencies of IFNγ^+^ NK cells were equivalent in Jα18^−/−^ and CXCR6^−/−^ Jα18^−/−^ mice reconstituted with iNKT cells (Fig. 6C).

### CXCL16-expressing DCs mediate enhanced tumor control and reduced liver toxicity

**Figure 7.**
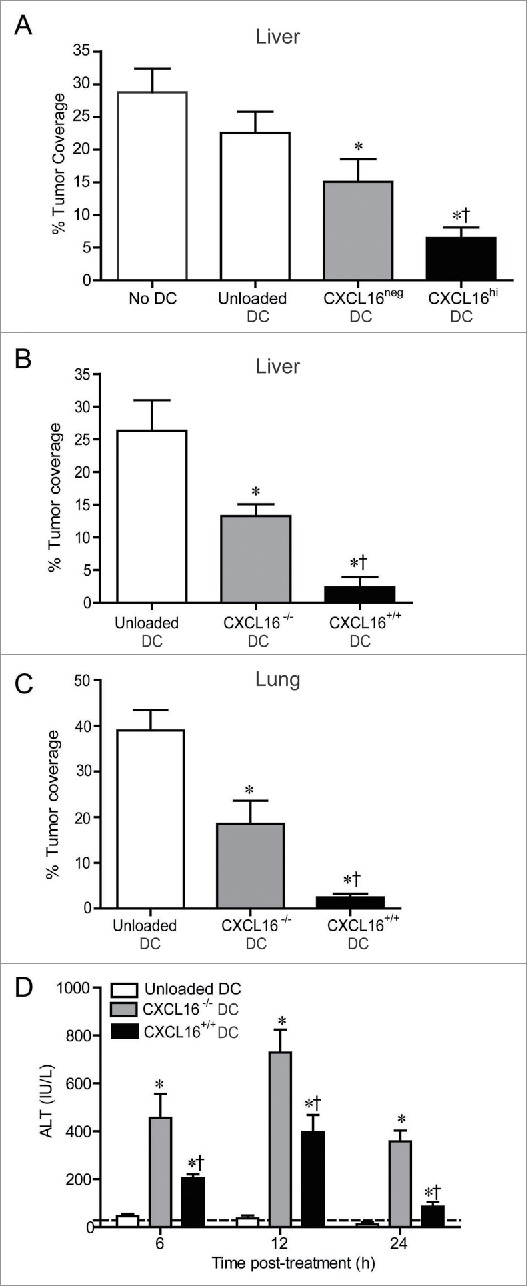
Control of metastatic B16 melanoma lesions in the liver and lung via transfer of glycolipid-loaded DCs. (A) To induce liver metastasis, wild-type mice were inoculated in the spleen with 2.5 × 10^5^ B16 melanoma cells. Five days later, mice were injected i.v. with 2 × 10^5^ α-GalCer-loaded CXCL16^hi^ or CXCL16^neg^ BMDCs, or unloaded control BMDCs generated from wild-type mice (n = 14–20 per group). *p < 0.05 compared to unloaded DCs, †*p* < 0.05 compared to CXCL16^neg^ DCs. (B) In independent experiments, 2 × 10^5^ α-GalCer-loaded BMDCs generated from wild-type or CXCL16^−/−^ mice were delivered 5 d after B16 inoculation (n = 9–10 per group). (C) To induce lung metastasis, wild-type mice were inoculated i.v. with 2.5 × 10^5^ B16 melanoma cells. Three days later, mice were injected i.v. with 2 × 10^5^ α-GalCer-loaded BMDCs generated from wild-type or CXCL16^−/−^ mice (n = 4–6 per group). Liver and lung metastasis were examined 14 d after tumor cell inoculation using image analysis software to calculate tumor coverage. (D) Serum ALT levels were measured in tumor-bearing mice following administration of wild-type or CXCL16^−/−^ DCs (n = 4–6 per group). *p < 0.05 compared to unloaded DCs, †*p* < 0.05 compared to CXCL16^−/−^ DCs.

Since potent IFNγ production is associated with enhanced antitumor responses,^11,12,19^ we examined whether CXCL16 co-stimulation of iNKT cells during glycolipid activation modified tumor clearance in an experimental model of B16-F10 melanoma metastasis to the liver.^4,8,35^ Wild-type BMDCs were loaded with α-GalCer and sorted into CXCL16^hi^ and CXCL16^neg^ populations prior to adoptive transfer into tumor-bearing mice. Administration of CXCL16^hi^ DCs mediated enhanced protection from B16 melanoma metastasis compared to CXCL16^neg^ DCs, unloaded DCs, or saline vehicle controls (Fig. 7A). Similarly, adoptive transfer of glycolipid-loaded CXCL16^+/+^ BMDCs mediated enhanced protection from tumor metastasis compared to CXCL16^−/−^ BMDCs (Fig. 7B). Given the high frequency of iNKT cells in the liver relative to other tissues, we wanted to determine whether CXCL16^+/+^ DCs could enhance tumor control at other sites. Administration of glycolipid-loaded CXCL16^+/+^ BMDCs also provided enhanced protection from B16 metastasis to the lung (Fig. 7C). These results demonstrate a critical role for CXCL16 in iNKT cell-mediated antitumor responses. Interestingly, treatment of tumor-bearing mice with CXCL16^−/−^ DCs induced higher levels of serum alanine aminotransferase (ALT) compared to CXCL16^+/+^ DCs (Fig. 7D). These findings are consistent with a protective role for IFNγ against α-GalCer-induced liver injury.^36^

## Discussion

iNKT cell activation mediated by presentation of glycolipid antigens has been shown to enhance antitumor immune responses in preclinical cancer models^4-7,9,19^ and clinical trials.^12,37-39^ Coordinated interactions between iNKT cells and glycolipid-presenting DCs can influence cytokine production and polarization of subsequent immune responses.^10,40^ In this study, we have demonstrated that co-stimulatory CXCR6/CXCL16 interactions specifically increase IFNγ production following glycolipid activation and lead to enhanced tumor control *in vivo* following adoptive DC transfer. These studies suggest that immunotherapies utilizing α-GalCer-pulsed CXCL16^hi^ DCs have the potential to induce superior antitumor responses in cancer patients compared to previous clinical trials in which bulk populations of glycolipid-loaded antigen-presenting cells were transferred.

CXCR6/CXCL16 interactions have been shown to influence cytokine production and immune responses in several studies. Th1 and Th17 polarization were impaired in collagen-immunized CXCR6^−/−^ mice, resulting in decreased development of collagen-induced arthritis.^41^ Similarly, CXCL16 blockade reduced serum IFNγ levels and *in vitro* antigen recall responses in a mouse model of multiple sclerosis.^42^ CXCL16 blocking antibodies also reduced IFNγ production and increased bacterial load in a mouse model of *Salmonella enterica* infection.^43^ The decrease in intracellular IFNγ production by CD3^+^ lymphocytes in this infection model could be related to impaired activation of CXCR6^+^ iNKT cells since *Salmonella typhimurium* glycolipids are known to stimulate iNKT cell responses.^2,44^ Consistent with our findings that CXCR6 on NK cells did not influence IFNγ production in response to glycolipid treatment, the use of CXCL16 blocking antibodies in the context of *S. enterica* infection did not alter the frequency of IFNγ-producing NK cells.^43^

CXCR6 is highly expressed on iNKT cells and has previously been shown to play important roles in iNKT cell trafficking, maturation, and cytokine production.^4,25,26,45^ However, the regulation of CXCL16 expression following administration of glycolipids has not been reported previously. Our results show a striking upregulation of CXCL16 on DCs following treatment with α-GalCer. This was dependent on crosstalk between iNKT cells and DCs as CXCL16 expression was not enhanced in iNKT cell-deficient Jα18^−/−^ mice. IFNγ, TNF, and IL-1α have been previously implicated in the upregulation of CXCL16 in other settings,^31,32^ suggesting that the diverse array of cytokines produced following iNKT cell activation (including TNF and IFNγ)^4,46^ may upregulate CXCL16 on DCs during glycolipid presentation.

Our current study demonstrates that immobilized CXCL16 or transmembrane CXCL16 on DCs can enhance the production of IFNγ from activated iNKT cells both *in vitro* and *in vivo*. This is consistent with, and extends, previous work demonstrating impaired IFNγ production from iNKT cells stimulated in culture with CXCL16^−/−^ DCs.^27^ Furthermore, we demonstrate that CXCL16 also enhances IFNγ production from activated human iNKT cells. IFNγ plays a role in diverse biological functions related to host defense and immune regulation in cancer. In particular, IFNγ upregulates MHC class I to increase antigen presentation,^47,48^ enhances polarization of Th1-mediated immune responses,^49^ and augments activation of NK cells.^50,51^ In addition, IFNγ has also been shown to have direct anti-proliferative,^52-54^ anti-angiogenic,^55,56^ and proapoptotic^57^ effects on cancer cells. In clinical studies, the benefits of iNKT cell activation therapy have been associated with the induction of IFNγ responses.^12^ Therefore, CXCR6/CXCL16 co-stimulatory interactions represent a potential target for manipulation in cancer therapies, where a boost in the Th1 IFNγ response is desired.

In addition to iNKT cells, CXCR6 is expressed on a subset of hepatic NK cells. While CXCR6 is dispensable for trafficking and retention of NK cells in the liver, it plays an important role in the function of hapten-specific memory NK cells.^34^ Given that NK cells are also potent producers of IFNγ following α-GalCer-induced iNKT cell activation,^8,11,19^ we examined the potential role of CXCR6/CXCL16 signals in modifying the response of NK cells to α-GalCer-pulsed CXCL16^hi^ DCs. Our results using iNKT cell reconstituted mice demonstrate that CXCR6 on NK cells (and other cell populations) does not play a direct role in enhancing IFNγ production in response to glycolipid-pulsed CXCL16^hi^ DCs. This suggests that the role of CXCR6/CXCL16 in regulating IFNγ production is directly linked to iNKT cell activation. Indeed, CXCL16^+/+^ DCs induced a larger number of IFNγ-producing iNKT cells and expansion of T-bet^+^ iNKT cells. This is consistent with preferential stimulation of the recently characterized Th1-like iNKT subset (iNKT1), that upregulates T-bet during thymic development and generates high levels of IFNγ.^58^ However, we cannot exclude the possibility that CXCR6/CXCL16 interactions also shift polarization of other iNKT cell subsets.

While increased IFNγ production by iNKT cells likely contributes to enhanced tumor control, iNKT cells do not need to generate IFNγ to mediate antitumor responses. Indeed, we have demonstrated that tumor control is normal in Jα18^−/−^ mice reconstituted with IFNγ^−/−^ iNKT cells.^8^ In these mice, intact transactivation of NK cell IFNγ responses was likely sufficient to mediate tumor control. Therefore, although CXCR6/CXCL16 interactions enhance beneficial IFNγ responses from iNKT cells, there could be additional mediators or processes regulated by CXCR6/CXCL16 interactions that contribute to tumor control.

In addition to the expression of CXCL16 on DCs, macrophages, and B cells,^28,29^ CXCL16 is expressed by a variety of mouse and human cancer cells.^59-61^ However, the role of CXCL16 in cancer is ambiguous. In a mouse model of colorectal cancer, CXCL16 inhibited liver metastasis via recruitment of CXCR6 expressing T cells and iNKT cells.^62^ CXCL16 expression is also strongly correlated with increased tumor infiltration of CD4^+^ and CD8^+^ T cells in colorectal cancer,^60^ and improved survival in cancer patients.^60,61,63^ While these beneficial effects may be related to transmembrane CXCL16, soluble CXCL16 has been linked to increased tumor metastasis.^64-66^ In colorectal cancer patients, high preoperative levels of serum CXCL16 were associated with recurring liver metastasis and a poor prognosis.^67^ Metalloproteinases ADAM-10 and ADAM-17, which cleave membrane CXCL16 to generate soluble CXCL16, have also been linked to increased tumor burden.^66,68,69^ Indeed, inhibition of ADAM10/17-mediated CXCL16 shedding reduced the migrating potential of ovarian cancer cells.^66^ Furthermore, soluble CXCL16 derived from myeloid-derived suppressor cells,^70^ or recombinant CXCL16,^71^ has been shown to play a role in angiogenesis. Taken together, these studies suggest that transmembrane CXCL16 may promote antitumor responses whereas soluble CXCL16 may contribute to tumor progression.

Although the distribution of iNKT cells is similar in humans and mice, the frequency of iNKT cells is ∼10-fold lower in humans, with significant variation between individuals.^23,72^ This has led to concerns that therapeutic studies in mice may not translate well to patients. However, mice containing human CD1d are protected from tumor metastasis via α-GalCer treatment, despite having iNKT cell frequencies comparable to humans.^73^ Similarly, we have shown that CXCR6^−/−^ mice, which have significantly reduced numbers of iNKT cells in the liver and lung, are protected from tumor metastasis by glycolipid treatments.^4^ These findings suggest that small numbers of iNKT cells can induce potent responses. To address the low frequency of iNKT cells in humans, some studies have treated cancer patients with *ex vivo* expanded iNKT cells in conjunction with glycolipid-loaded DCs, with beneficial results.^38,39^ We have observed beneficial effects of adoptive iNKT cell transfer in iNKT cell deficient mice^8^ but not wild-type mice,^5^ which may be expected if the endogenous iNKT cells in wild-type mice exceeds the number needed for protective responses.

In conclusion, our data demonstrate that CXCR6/CXCL16 co-stimulatory interactions between DCs and iNKT cells play an important role in mediating glycolipid-dependent tumor control. DCs upregulate CXCL16 during glycolipid stimulation, leading to enhanced IFNγ production from iNKT cells. Adoptive transfer of glycolipid-loaded CXCL16^hi^ DCs enhanced IFNγ production and tumor control, while inducing less liver toxicity, compared to CXCL16^neg^ DCs. Our findings provide preclinical evidence to support the clinical use of glycolipid-loaded CXCL16^hi^ DCs to enhance outcomes in cancer patients undergoing iNKT cell activation immunotherapy.

## Materials and methods

### Mice

C57BL/6J mice were obtained from the Jackson Laboratories. Jα18^−/−^mice were generated in the laboratory of Dr M. Taniguchi (RIKEN Research Center for Allergy and Immunology).^74^ CXCR6^−/−^ mice containing an enhanced green fluorescent protein replacement were obtained from Dr D. Littman (New York University Medical Center).^75^ CXCL16^−/−^ mice were generated at the National Institutes of Health (NIH) Mutant Mouse Regional Resource Center (strain 032260-UCD; University of California, Davis) using targeted embryonic stem cells donated by Genentech Inc. All strains were backcrossed on the C57BL/6 background for at least 12 generations prior to use in experiments. CXCR6^−/−^Jα18^−/−^ double knockout mice were generated in house by crossing CXCR6^−/−^and Jα18^−/−^ mice. Mice were housed within the Carleton Animal Care Facility at Dalhousie University and used between 6 and 12 weeks of age. Experiments were performed with approval from the University Committee on Laboratory Animals following guidelines set by the Canadian Council on Animal Care.

### Cell lines

B16-F10 melanoma cells (American Type Culture Collection) were cultured at 37°C, 5% CO_2_, in Dulbecco's Modified Eagle Medium supplemented with 10% fetal bovine serum, 100 µg/mL streptomycin, and 100 U/mL penicillin (Fisher-Hyclone). Cells were harvested in the logarithmic growth phase using trypsin-ethylenediaminetetraacetic acid treatment (Sigma-Aldrich). Washed cells were re-suspended in saline for use in tumor metastasis experiments.

### Glycolipid stimulation

To examine changes in CXCL16 expression on antigen-presenting cells, mice were injected intraperitoneally with 4 µg of α-GalCer [(2S,3S,4R)-1-*O*-(α-D-galactopyranosyl)-2-(*N*-hexacosanoylamino)-1,3,4-octadecanetriol] purchased from Toronto Research Chemicals.

### Flow cytometry/antibodies

The following antibody clones were used for cell staining: Fluorescein isothiocyanate (FITC)-conjugated TCRβ (clone H57-597), CD11c (clone Hl3), NK1.1 (clone PK136), and B220 (clone RA3-6B2); Phycoerythrin (PE)-conjugated IL-4 (clone 11B11), IFNγ (clone XMG1.2), CD40 (clone 1C10), CD80 (clone 16-10A1), CD86 (clone GL1), CD1d (clone 1B1), and NK1.1 (clone PK136); PerCP-Cychrome 5.5-conjugated NK1.1 (clone PK136), CD19 (clone 1D3), CD11b (clone M1/70), T-bet (clone eBio4B10); allophycocyanin (APC)-conjugated CD86 (clone GL1), CD11b (clone M1/70), B220 (clone RA3-6B2), and F4/80 (clone BM8) were purchased from eBioscience. PE-conjugated CD154 (clone MR1), CD69 clone (H1.2F3), and I-A (clone M5/114.15.2); PerCP-Cychrome 5.5-conjugated CD3 (clone 145-2C11), CD19 (clone 1D3) and streptavidin; and APC-conjugated CD11c (clone N418) were purchased from BD Biosciences. A biotin-conjugated polyclonal CXCL16 antibody was purchased from PeproTech Inc. PE- and APC-labeled streptavidin and the nucleic acid dye 7-aminoactinomycin D (7-AAD) were purchased from eBioscience. CXCR6 expression was detected using a chimeric CXCL16-Fc fusion protein,^20^ followed by a PE-conjugated goat anti-human Fcγ polyclonal antibody (Jackson ImmunoResearch Laboratories). APC-conjugated CD1d tetramers loaded with the α-GalCer analog PBS57 were obtained from the NIH Tetramer Core Facility (Emory Vaccine Center at Yerkes). For labeling human iNKT cells, FITC-TCRβ (clone IP26) and PE-TCR-Vα24Jα18 (clone 6B11) antibodies were used (eBioscience). Flow cytometry was performed using a two laser FACSCalibur with BD CellQuest Pro software (BD Biosciences). Isotype-matched control antibodies were used for all analyses. Intracellular cytokine staining was performed using a BD Cytofix/Cytoperm kit (BD Biosciences).

### Cell isolation

Liver and spleen lymphocytes were isolated by mechanical dispersion through a 70 µm wire mesh. Following dissociation into single-cell suspensions, liver lymphocytes were separated from hepatocytes by centrifugation through a 33% isotonic Percoll gradient (GE Healthcare). Red blood cells were lysed with ammonium chloride buffer (150 mM NH_4_Cl, 10 mM KHCO_3_, and 0.1 mM EDTA) and cells were washed prior to use.

BMDCs were generated as previously described.^5^ Non-adherent cells were isolated from bone marrow cultures on day 6 and loaded overnight in complete Roswell Park Memorial Institute-1640 media (containing 10% fetal bovine serum, 50 mM 2-mercaptoethanol, 2 mM L-glutamine, 100 µg/mL streptomycin, 100 units/mL penicillin) supplemented with 20 ng/mL recombinant mouse GM-CSF (PeproTech) and 200 ng/mL α-GalCer.

### Mouse cell sorting

Liver mononuclear cells were isolated as described above. Prior to staining, cells were pre-incubated with anti-CD16/32 (clone 97) to block Fc-receptors. iNKT cells were sorted as TCRβ^+^ CD1d tetramer^+^ cells. To isolate DC populations from bulk splenocytes, FITC-CD11c^+^ cells were purified with anti-FITC microbeads by MACS Cell Separation (Miltenyi Biotech). CD11c^+^ cells were loaded with glycolipid by culturing at 37°C for 24 h in Roswell Park Memorial Institute-1640 media containing 10% fetal bovine serum, 10 ng/mL of GM-CSF, and 200 ng/mL α-GalCer or loading vehicle. Cells were stained with antibodies against NK1.1, CD11c, CXCL16, and 7-AAD (and CD86 for the CD86^hi^ transfer experiments) for sorting. Sorted CXCL16^hi^ DCs were 7AAD^−^ NK1.1^−^ CD11c^+^ CXCL16^hi^ (and in some cases CD86^hi^) and sorted CXCL16^neg^ DCs were 7-AAD^−^ NK1.1^−^ CD11c^+^ CXCL16^neg^ (and in some cases CD86^hi^). In other experiments, 7AAD^−^ NK1.1^−^ CD11c^+^ splenocytes were sorted from wild-type or CXCL16^−/−^ mice. All cells were sorted using a BD FACSAria sorter with BD FACSDiva software (BD Biosciences).

### DC and iNKT cell co-culture

CD1d-tetramer^+^ iNKT cells were purified by flow sorting and cultured with α-GalCer-loaded CXCL16^hi^ or CXCL16^neg^ splenic DCs at a ratio of 1:2, DCs to iNKT cells. A total of 40,000 cells were cultured in 96-well tissue culture plates. Supernatants were harvested after 24 h for cytokine analysis.

### Generation of primary human iNKT cell lines

Isolation of peripheral blood mononuclear cells from healthy human volunteers was done under institutional ethics approval to the REACH Team (Dalhousie University). Blood was collected in endotoxin-free sodium heparin tubes and PBMCs were isolated using Lymphoprep (Stem Cell Technologies). PBMCs were washed twice prior resuspension in complete RPMI-1640 (supplemented with 100 µg/mL streptomycin, 100 U/mL penicillin, 10% autologous human plasma), containing recombinant human IL-2 (10 ng/mL), and recombinant human IL-15 (5 ng/mL) (Peprotech). Blood iNKT cells were expanded in 6-well plates for one week using 200 ng/mL α-GalCer and TCRβ^+^Vα24Jα18^+^ iNKT cells were sorted.

### *In vitro* anti-CD3 stimulation of iNKT cells

Mouse liver mononuclear cells were cultured in 96-well plates coated with anti-CD3 (clone 145-2C11), with or without 100 ng/mL of recombinant extracellular domain mouse CXCL16 (R&D Systems). After 2 h, intracellular cytokine staining for IFNγ and IL-4, and surface staining for CD40L and CD69 were analyzed on CD1d-tetramer^+^ TCRβ^+^ iNKT cells by flow cytometry. For stimulation of human iNKT cells, 5 × 10^4^ sorted cells were cultured in anti-CD3- (clone OKT3) coated wells with or without 100 ng/mL recombinant extracellular domain human CXCL16 (BioLegend). Supernatants were collected 24 h following stimulation for analysis of IFNγ and IL-4 levels by enzyme-linked immunosorbent assay (ELISA) (eBioscience).

### iNKT cell activation by adoptive DC transfer

Splenic DCs isolated from wild-type mice were loaded overnight with α-GalCer (200 ng/mL) and sorted into CXCL16^hi^ and CXCL16^neg^ subsets. DCs were injected i.v. (4 × 10^4^ per recipient) into C57BL/6 or Jα18^−/−^ mice. As controls, unloaded DCs or saline vehicle were injected into recipient mice. Serum samples were collected at 2, 6 and 18 h after transfer. These experiments were repeated using purified splenic DCs or cultured BMDCs from wild-type versus CXCL16^−/−^ mice. In separate experiments, iNKT cells were isolated 2 or 72 h following administration of α-GalCer loaded CXCL16^+/+^ versus CXCL16^−/−^ DCs to examine intracellular IFNγ and T-bet expression.

### Adoptive transfer of LPS pulsed CXCL16^+/+^ or CXCL16^−/−^DCs

To examine whether LPS pulsed DCs would stimulate IFNγ production *in vivo*, CXCL16^+/+^ or CXCL16^−/−^ BMDCs were treated with 250 ng/mL LPS for 4 h. BMDCs were washed 5 times prior to i.v. adoptive transfer into naive mice. Serum samples were collected at 6, 12, 24, 48 and 72 h to examine IFNγ levels by ELISA.

### Cytokine array analysis

A MULTI-SPOT 96-well custom mouse cytokine array with 10 analytes (GM-CSF, IFNγ, IL-1β, IL-2, IL-4, IL-5, IL-6, IL-10, IL-12p70, TNF) was used to measure cytokine levels in serum and culture supernatants (Meso Scale Discovery). Plates were read with a Sector Imager 2400 and analyzed with Discovery Workbench software (Meso Scale Discovery). In some experiments, mouse IL-4, IFNγ, and IL-12p70 were measured using Ready-SET-Go! ELISA kits (eBioscience).

### *In vivo* iNKT cell expansion and reconstitution

To investigate the role of CXCR6/CXCL16 interactions on NK cell function, sorted iNKT cells were reconstituted into Jα18^−/−^ and CXCR6^−/−^Jα18^−/−^ mice.^8^ iNKT cells were expanded in donor mice by i.v. transfer of 6 × 10^5^ α-GalCer-loaded bone marrow DCs. After 72 h, liver mononuclear cells were isolated and 1 × 10^7^ TCRβ^+^CD1d tetramer^+^iNKT cells were adoptively transferred into Jα18^−/−^ and CXCR6^−/−^Jα18^−/−^ mice. Twenty-four hours following reconstitution, recipient mice received i.v. 2 × 10^5^ α-GalCer-loaded CXCL16^hi^ BMDCs. Serum and intracellular IFNγ were monitored 18 h following stimulation.

### Liver and lung B16-F10 melanoma metastasis models

To study liver metastasis, B16-F10 melanoma cells (2.5 × 10^5^) were aseptically inoculated into the spleen of wild-type mice.^4,8,76^ Five days later, mice were treated i.v. with 2 × 10^5^ α-GalCer-loaded CXCL16^hi^ or CXCL16^neg^ BMDCs, or unloaded control BMDCs generated from wild-type mice. In separate experiments, α-GalCer-loaded BMDCs from wild-type or CXCL16^−/−^ mice were transferred. To examine lung metastasis, mice were inoculated i.v. with 2.5 × 10^5^ melanoma cells.^76^ Three days later, mice were treated i.v. with α-GalCer loaded CXCL16^+/+^ or CXCL16^−/−^ DCs. Fourteen days after tumor cell injection, mice were sacrificed and images of the anterior and posterior surface of each liver/lung were acquired using a Micropublisher 3.3 digital camera with QCapture (v.2.8.1) software (QImaging).^8,76^ The relative tumor coverage on the anterior and posterior surfaces was analyzed using Image J software (NIH).

### Liver toxicity

To monitor liver toxicity following iNKT cell activation with α-GalCer loaded CXCL16^+/+^ or CXCL16^−/−^ DCs, serum samples were collected from tumor-bearing mice 6, 12 and 24 h after DC transfer. Levels of alanine aminotransferase in the serum were quantified by NADH oxidation assay (Cayman Chemicals).^5^

## Statistical analysis

Data are expressed as mean ± SEM unless otherwise stated. A non-parametric two-tailed Mann–Whitney test was used to compare between two data groups. Comparisons between more than two data groups were made using a Kruskal–Wallis non-parametric ANOVA with Dunn's post-test. Statistical significance was set at *p* < 0.05.
